# Innovations in Diagnostic Imaging in Oral and Maxillofacial Diseases

**DOI:** 10.3390/diagnostics12020536

**Published:** 2022-02-19

**Authors:** Maurilio D’Angelo, Alessio Zanza, Dario Di Nardo, Luca Testarelli

**Affiliations:** Department of Oral and Maxillofacial Sciences, La Sapienza University of Rome, 00161 Rome, Italy; maurilio.dangelo@uniroma1.it (M.D.); dario.dinardo@uniroma1.it (D.D.N.); luca.testarelli@uniroma1.it (L.T.)

In recent years, improvements in imaging techniques have profoundly facilitated the diagnosis of pathologies of the maxillofacial district. Three-dimensional radiographic diagnostic exams, analyzed by software that allow easy viewing of images and various graphic reworkings, are frequently applied to dentistry and the maxillofacial district for the diagnosis and treatment of pathologies and conditions that, until a few years ago, required several radiographic examinations [1,2,3]. CBCT today represents the most widespread and used 3D exam in dentistry, given its presence and availability in dental offices. The ability to modify the FoV while maintaining a very high image quality ranges from use in the smallest FoVs for a few teeth in endodontics to larger FoVs, for example, in orthodontics [3].

It is precisely because of its usefulness in orthodontics and orthognathic surgery with large FOVs, in oral surgery and implantology for the 3D evaluation of bone volumes and proximity to noble structures, in endodontics for the understanding of the often-difficult root canal system anatomy with reduced FOV and greater resolution that this imaging technique is widely used. [3,4,5].

This method allows to modify many parameters and different aspects allowing easy use, speed, and optimization of radiation for diagnostic purposes, often decisive in differential diagnoses, and to facilitate surgical–endodontic treatment in aid of guided or navigated surgeries [6,7,8,9].

In this regard, the patient’s exposure to a radiation dose that can cause biological damage must always be kept in mind, and for this reason, there is a growing interest in MRI (magnetic resonance imaging), for imaging techniques that use ultrasound. The clinical dental uses of both are increasingly investigated, although the ultrasound examination is operator-dependent, unlike magnetic resonance imaging, which today turns out to be a more complex examination due to the long data acquisition times for having particularly high resolutions [10,11,12].

Recent literature highlights how it is possible to consider MRI as a complete dental diagnostic examination, which allows for both an investigation of the anatomy of the soft tissues at certain frequencies and the volumes and bone density [10]. In this regard, recently published evidence shows that MRI is superimposable, in the planning of implant surgery, to the static or dynamic guided surgery planned using CBCT [10,11]. This represents a major step towards radiation-free diagnostics, which are increasingly innovative and protective of the patient [12].

Regarding the ultrasounds and their physical characteristics, their application in dentistry is increasingly studied, historically used in gnathology, considering that the superficiality of the other structures to be studied is extremely valid. The mucous tissues of the oral cavity, or the supporting bone, with the appropriate probes seem to be simple to apply, free of ionizing radiation, minimally invasive, and easily available in the office for constant use during daily clinical practice [13,14].

Unlike resonance, ultrasound allows for a more specific evaluation of a superficial mucous or bone site, with specific parameters capable of allowing a detailed analysis. With these specific parameters, this examination also allows for the evaluation of dental hard tissues, which has not been explored with this method to date. The growing interest in this technique could lead in the future to avoiding the overuse of intraoral periapical or bite-wing radiographs [14].

Future in vitro and in vivo studies will be needed to ascertain the effectiveness and innovation brought about by the application of these techniques. The instruments for oral use will have to be implemented, especially the probes for examinations with ultrasound and acquisition times for MRI [13,14,15]. The margins of success are wide, guided by the non-invasiveness of these procedures and by the absence of biological damage caused by ionizing radiation.

## Data Availability

This study reports any data.
